# Fulminant and fatal *Trypanosoma cruzi* reactivation in a patient with lymphoma

**DOI:** 10.1128/asmcr.00157-25

**Published:** 2025-11-17

**Authors:** Georgi Lukose, Anna Mertelsmann, Rebecca M. Marrero Rolon, David J. Pisapia, Sanjay Patel, Christina Coyle, Lars F. Westblade, Michael Satlin, Jamie Marino

**Affiliations:** 1 Department of Pathology and Laboratory Medicine, Weill Cornell Medicine 207092 , New York, New York, USA; 2 Department of Infectious Diseases and Hospital Epidemiology, University Hospital Zurich 27243 , Zurich, Switzerland; 3 University of Zurich 27217, Zurich, Switzerland; 4 Department of Medicine, Division of Infectious Diseases, Weill Cornell Medicine 12295, New York, New York, USA; 5 Department of Medicine, Albert Einstein College of Medicine 200531, New York, New York, USA; 6 Department of Pediatrics, Division of Infectious Diseases, Weill Cornell Medicine 205138 , New York, New York, USA; Pattern Bioscience, Austin, Texas, USA

**Keywords:** *Trypanosoma cruzi*, reactivation, Chagas disease, lymphoma

## Abstract

**Background:**

*Trypanosoma cruzi* is a protozoan parasite and the causative agent of Chagas disease. This case describes a fulminant and fatal reactivation of *T. cruzi* after immunosuppression in a patient with a history of follicular lymphoma, with parasitemia detectable in peripheral blood smears and parasites seen in multiple autopsy tissue specimens.

**Case Summary:**

A 61-year-old woman who had immigrated from El Salvador and was receiving obinutuzumab and zanubrutinib for follicular lymphoma in remission was admitted with persistent COVID-19 pneumonia. After multiple therapies for COVID-19, including more than 3 weeks of corticosteroid therapy for the possibility of organizing pneumonia, a peripheral blood smear identified *T. cruzi* trypomastigotes, and later amastigotes were identified in a bone marrow biopsy. The patient was treated with benznidazole but ultimately died. At autopsy, amastigotes were observed in multiple organs, including the heart, esophagus, stomach, small intestine, colon, bladder, and skeletal muscle.

**Conclusion:**

Most cases of Chagas reactivation are described in people living with HIV-1 or transplant recipients. Rarely, *T. cruzi* reactivation can occur in patients undergoing immunosuppressive therapies for malignancies or inflammatory states like COVID-19 infection, as seen in this case. If not recognized early, reactivation can be fatal despite antiparasitic treatment. Providers should consider screening patients from endemic areas who will start on immunosuppressive therapies. Repeating screening may be of value with periods of new immunosuppression. In patients with Chagas disease and malignancy, PCR from blood should be performed during enhanced immunosuppression so that preemptive treatment can be initiated prior to the presentation of fulminant disease.

## INTRODUCTION

Chagas disease, also known as American trypanosomiasis, is a disease caused by infection of the protozoan parasite *Trypanosoma cruzi* (1–3). Considered a neglected tropical disease, it affects an estimated 6 million people in Latin America (4). Due to increased migration, it has become a global health concern with approximately 288,000 infected persons in the United States, many of whom remain undiagnosed (3–7). *Trypanosoma cruzi* results in a lifelong infection and can establish long-term persistence (chronic infection) when untreated, where 20%–30% of cases will progress to severe cardiac or gastrointestinal disease (1, 3, 8). Patients with chronic Chagas disease with significant dysfunction of one or more immune mechanisms can have uncontrolled replication of the parasite, leading to high levels of parasitemia with severe complications (6, 9). Reactivation is most frequently reported in people with HIV-1 with low CD4 cell counts and those individuals undergoing immunosuppressive therapy after transplant, but it is less commonly reported in malignancy (6, 7, 9). In this report, we describe a case of a fulminant and fatal reactivation of Chagas disease in a patient who had immigrated to the United States to highlight the importance of considering reactivation during periods of increased immunosuppression.

## CASE PRESENTATION

The patient was a 61-year-old woman with a history of follicular lymphoma, which was diagnosed 5 years previously when she had presented with a retroperitoneal mass. The patient’s lymphoma had initially responded to chemotherapy. Two years prior to the current presentation, the patient had relapsed disease with bone marrow involvement, leading to chimeric antigen receptor T (CAR-T) cell therapy with axicabtagene ciloleucel. The disease relapsed 8 months after CAR-T cell therapy. The patient was then treated with obinutuzumab and zanubrutinib, achieving a complete response. The patient was in remission at the time of this presentation.

While receiving maintenance obinutuzumab and zanubrutinib and valacyclovir prophylaxis, the patient developed fever and cough and was hospitalized for COVID-19 pneumonia that was treated with remdesivir (100 mg every 24 hours, intravenously), dexamethasone, and antibacterial therapies. Obinutuzumab and zanubrutinib were held in this setting. Two weeks after discharge, the patient was readmitted to our hospital with fever up to 38.9°C, dyspnea on exertion, a persistent non-productive cough, and diarrhea. A nasopharyngeal swab sample tested for SARS-CoV-2 by PCR showed a persistent positive result. Computed tomography of the chest showed increased multifocal ground-glass opacities predominantly involving the left upper lobe and bilateral lower lobes. The patient was started on empiric antimicrobial therapy with piperacillin-tazobactam (4.5 g every 8 hours, intravenously) and vancomycin (1.0 g every 8 hours, intravenously) on admission. Trimethoprim/sulfamethoxazole prophylaxis (800 mg/160 mg, three times a week, orally) was also initiated in the setting of ongoing corticosteroid therapy for severe COVID-19.

Initial microbiologic workup included blood cultures, sputum culture, urine *Legionella* antigen, serum (1→3)-β-D-glucan, and aspergillus galactomannan testing, all of which were negative. The patient was negative for human immunodeficiency virus by a fourth-generation antigen/antibody test and had previously had a negative interferon-gamma release assay test for *Mycobacterium tuberculosis* and a negative IgG for *Strongyloides* spp.

The patient remained febrile despite antimicrobial therapies and developed worsening hypoxic respiratory failure, requiring oxygen supplementation via high-flow nasal cannula. This led to treatment with corticosteroids, tocilizumab, remdesivir (100 mg every 24 hours, intravenously), and baricitinib. The patient eventually defervesced, and the hypoxia moderately improved but was still requiring 5 L of oxygen supplementation via nasal cannula. The patient continued to test positive for SARS-CoV-2 by PCR. Two weeks after admission, while continuing to receive daily methylprednisolone, the patient developed leukopenia, thrombocytopenia, and increased liver enzymes and lipase levels.

**Fig 1:**
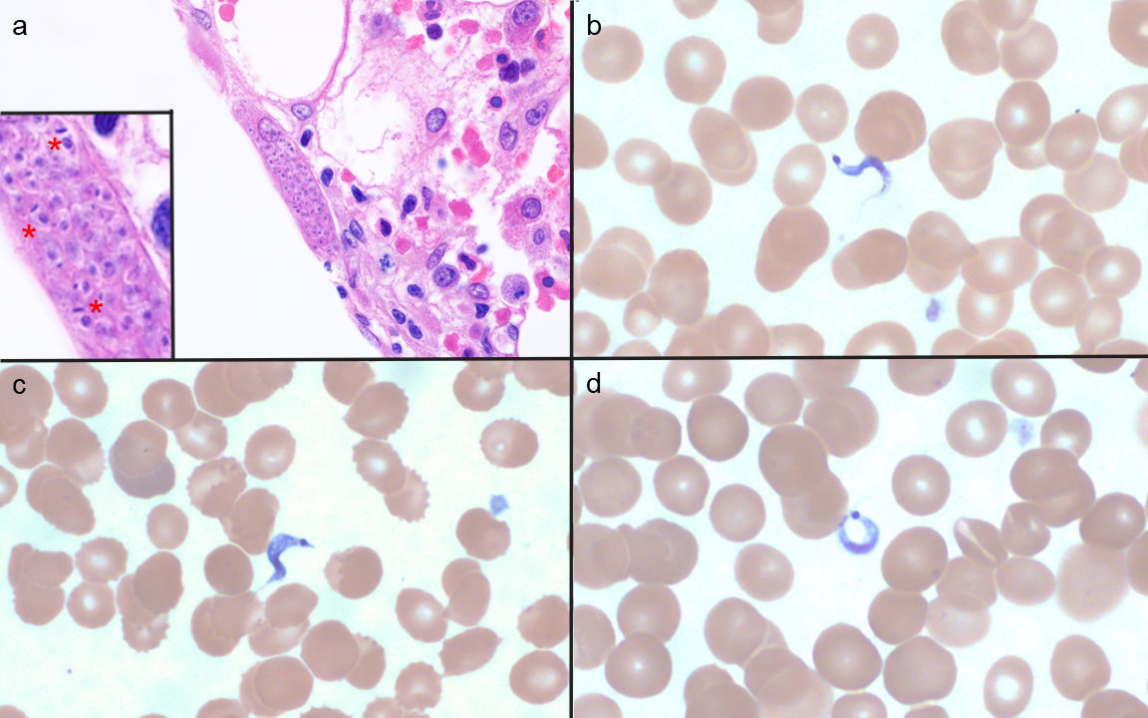
Antemortem bone marrow biopsy with a nest of *T. cruzi* amastigotes (a, ×1,000 magnification). Amastigotes with a nucleus and kinetoplast are visualized (**a**, inset *). Antemortem peripheral blood smear, stained with Wright-Giemsa, with *T. cruzi* trypomastigotes (**b–d**, ×1,000 magnification and digitally magnified 4x). Trypomastigotes have a subterminal to terminal large kinetoplast, central nucleus, undulating membrane, and flagellum.

A routine peripheral blood smear obtained on hospital day 26 showed *T. cruzi* trypomastigotes (Fig. 1). *T. cruzi* DNA was identified using PCR on a peripheral blood specimen. A bone marrow biopsy was performed, which showed *T. cruzi* amastigotes (Fig. 1). Further review of the patient’s history revealed that she was born and raised in rural El Salvador and moved to the United States over 30 years ago. A *T. cruzi* total antibody enzyme immunoassay performed prior to starting CAR-T cell therapy was reactive, but repeated peripheral blood smears and PCR for *T. cruzi* were negative at that time. There was no evidence of reactivation during therapy for lymphoma prior to this presentation. An electrocardiogram was performed, which showed sinus rhythm with a right bundle branch block. A high-sensitivity troponin I level was 3,126 ng/L (reference range [RR]: ≤40 ng/L) at the time of diagnosis, and a transthoracic echocardiogram showed an ejection fraction of 55%–60% with reduced right ventricular function. Benznidazole was obtained from the Centers for Disease Control and Prevention (CDC), and the patient was treated for 2 days with 62.5 mg twice daily, followed by 125 mg twice daily. The patient also received empiric treatment for *Strongyloides stercoralis* with ivermectin (12 mg daily for 2 days).

Despite treatment with benznidazole, the patient was intubated due to increasing oxygen requirements. A new transthoracic echocardiogram showed a global reduction of left ventricular function with an ejection fraction of 25%–30%, dilation of the right-sided chambers, reduced right ventricular function, and interatrial shunt. High-sensitivity troponin-I increased to 32,000 ng/L (RR: ≤40 ng/L). The patient became increasingly hypotensive despite maximal vasopressor support, and the patient died on hospital day 36.

**Fig 2:**
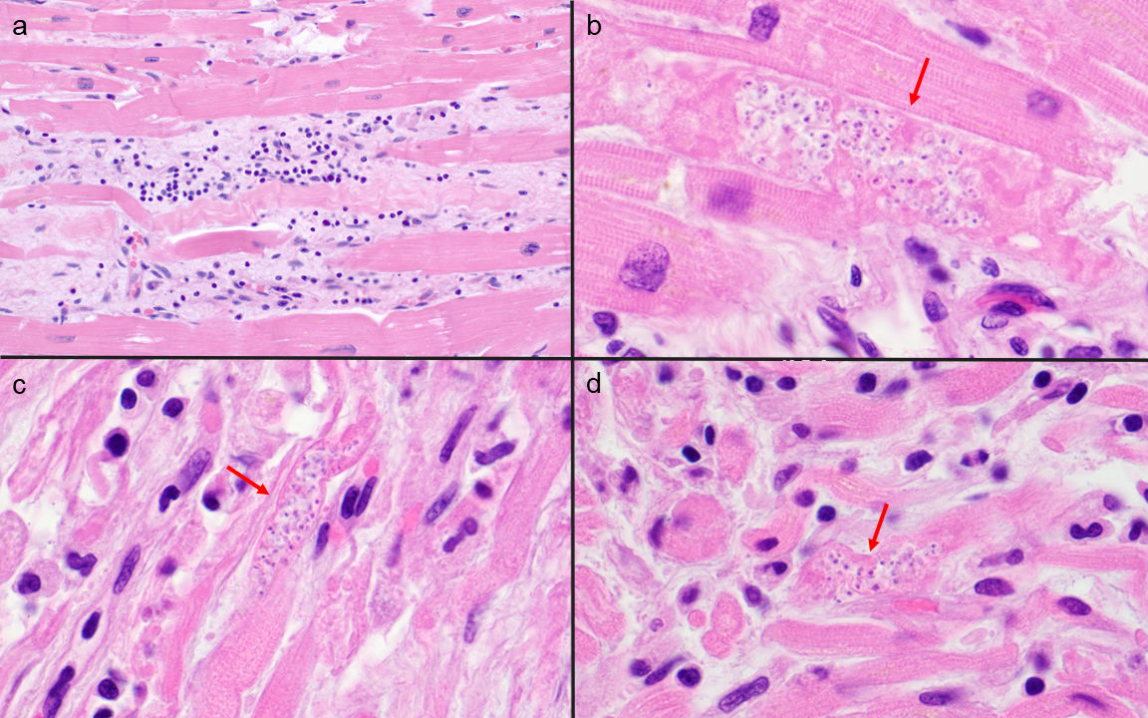
Postmortem examination of heart tissue demonstrated myocarditis with a chronic inflammatory infiltrate of lymphocytes and histiocytes (**a**, ×400 magnification). *T. cruzi* amastigotes (arrow) were observed in cardiomyocytes of the left ventricular myocardium (**b**, ×1,000 magnification), sinoatrial nodal region (**c**, ×1,000 magnification), and atrioventricular nodal region (**d**, ×1,000 magnification).

**Fig 3:**
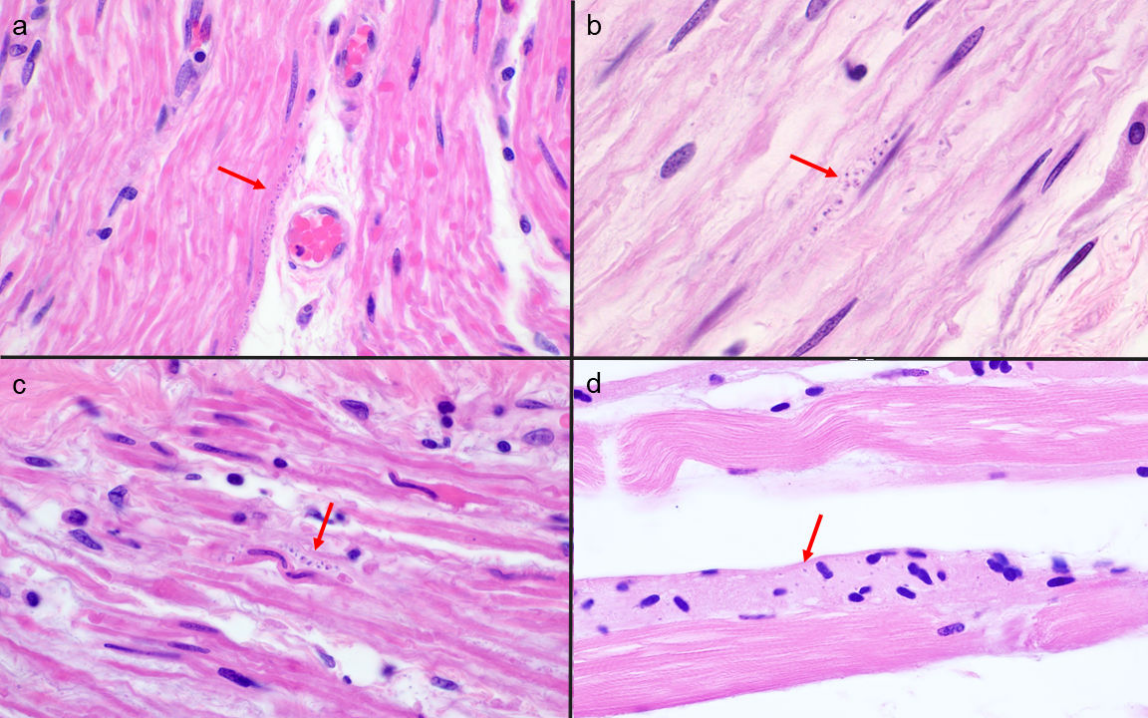
Postmortem examination demonstrated intracellular *T. cruzi* amastigotes (arrow) in smooth muscle cells of the muscularis of the esophagus (**a**, ×400 magnification), colon (**b**, ×1,000 magnification), and bladder (c, ×1,000 magnification). Intracellular *T. cruzi* amastigotes were also identified in skeletal muscle cells with injury or necrosis (**d**, ×1,000 magnification).

An autopsy was performed with appropriate consent from next of kin. Microscopic examination of hematoxylin and eosin-stained sections showed extensive myocarditis, with amastigotes identified within cardiomyocytes in multiple regions of the heart, including the bilateral ventricles, the right atrium (involving the regions of the sinoatrial node and atrioventricular node), and the interventricular septum (Fig. 2). A mixed inflammatory infiltrate of predominantly T lymphocytes and histiocytes was seen. Clusters of intracellular amastigotes were also identified in smooth muscle bundles of the muscularis of the esophagus, stomach, duodenum, ileum, colon, and bladder. Rare amastigotes were identified in skeletal muscle sampled from the quadriceps and psoas, where myonecrosis was seen (Fig. 3). The lungs showed diffuse alveolar damage with organization and hemorrhage. No evidence of residual follicular lymphoma was identified.

## DISCUSSION

Chagas disease is associated with low socioeconomic status, rural or jungle residences, and housing constructed from adobe, straw, or wood, which facilitate contact with triatomine bugs (family *Reduviidae*, subfamily *Triatominae*), the vector for *T. cruzi* (2, 5, 6, 10). Infection occurs when the triatomine bug takes a blood meal and its feces containing the parasite enter the bite site or the mucosal membranes of the mammalian host (6, 11). Less common routes of exposure include solid organ transplantation, blood transfusions, congenital transmission, and oral transmission via contaminated food and water (10). In endemic areas, infection is often acquired during childhood, where primary infection is usually asymptomatic, and in conjunction with a potential lack of consistent quality medical care, infections are usually not immediately identified, resulting in most individuals being untreated, leading to lifelong disease (6, 7). Due to migration, cases of Chagas disease have been reported throughout the world and are often represented by a highly vulnerable immigrant population, commonly without access to medical care or who are cared for by providers lacking expertise in Chagas disease (7). Common abnormalities found in Chagas cardiomyopathy include myocardial fibrosis and electrocardiographic changes, including right bundle branch blocks and arrhythmias, and heart failure (3, 8). This patient demonstrated electrocardiogram abnormalities including right bundle branch block. Digestive involvement is less common than cardiac but may involve symptoms such as megaesophagus or megacolon (3). Additionally, digestive involvement in Chagas disease is often only seen in individuals who have immigrated from countries south of the equator (3). This case describes a patient who had chronic *T. cruzi* cardiac disease, on immunosuppressive therapy, with fulminant reactivation while receiving maintenance obinutuzumab and zanubrutinib for lymphoma as well as corticosteroid therapy for COVID-19 pneumonia.

Diagnosis of chronic Chagas disease often relies on serology because the parasite burden is typically insufficient for detection on routine peripheral blood smear (12–14). There are a variety of serologic tests available, including enzyme-linked immunosorbent assay, indirect immunofluorescence, and indirect hemagglutination (6, 12, 13). Establishing the diagnosis of Chagas disease requires two positive serologic tests using different antigens (6). Screening for Chagas disease by serology is recommended for patients who have lived in Latin America, have family members who have lived in Latin America, or have been diagnosed with Chagas disease, and for all who have had contact with a triatomine vector (6). This diagnostic approach is different from screening for *Strongyloides stercoralis*, where two serologic tests are not a formal recommendation (15).

The mechanism of reactivation of *T. cruzi* is not well understood, but for those who are chronically infected, symptoms can be severe and often involve cardiac, dermatologic, and central nervous system (CNS) involvement (3, 8). The most frequent manifestations of reactivation in transplant patients are skin lesions, which usually appear as inflammatory nodules, and reactivation myocarditis, which on biopsy reveals histology similar to that seen in acute Chagas disease (9, 14). Conversely, patients with HIV-1 with lower CD4 counts are more likely to present with CNS disease (9).

Protocols for monitoring Chagas patients post transplantation recommend quantitative PCR (qPCR) for the first 6 months of immunosuppression and thereafter when immunosuppression is increased for transplant rejection, which reactivation can mimic (6, 9). The Infectious Disease Society of America recommends qPCR as the earliest and most sensitive indicator of reactivation, with sequential qPCRs required to monitor the rise in parasite load over time (6, 9). Prospective monitoring by qPCR is essential to detect and preemptively treat reactivation as delays in treatment of Chagas disease are associated with high mortality (6). In transplant patients, prospective monitoring by PCR has been shown to detect reactivation months prior to detecting parasites by microscopy, allowing for early treatment and excellent outcomes (6). If qPCR is unavailable, monitoring can be done by other methods such as direct microscopy; however, it is less sensitive as compared to qPCR (9).

Reactivation of Chagas disease in patients with neoplasm and immunosuppression is less commonly reported. Most reports have been in patients with leukemia or lymphoma, while there are no reports of reactivation of Chagas disease in patients with solid tumors. The role of prospective monitoring for reactivation of Chagas disease in the setting of lymphoma therapy while undergoing immunosuppressive regimens is less clear, and this case highlights a poor outcome associated with initiating treatment at a time when the organism was already evident on peripheral blood smear. In this case, the patient did not have any recorded reactivation or treatment during the lymphoma therapy. At the time of the reactivation, the patient was on maintenance therapy for lymphoma and high-dose steroids for COVID-19, where the steroids likely contributed to the reactivation of *T. cruzi*. This is further supported by prior case reports that have described Chagas disease reactivation in patients who were receiving immunosuppressive therapy, including high-dose steroids (16).

Nifurtimox and benznidazole are the only agents with proven efficacy against Chagas disease in the acute phase and in children under 18 years of age (3, 6, 7, 10, 14). A 60-day course is recommended for both agents, with benznidazole being the preferred first-line therapy as it is better tolerated and has more clinical trial data to support efficacy (6, 7). However, these agents have many adverse side effects as their mechanism of action is to generate toxic metabolites to disrupt parasite metabolic pathways (3, 6, 10, 14). In the BENEFIT trial, the only multicenter randomized, placebo-controlled trial in patients with cardiomyopathy, benznidazole had no significant benefit in preventing the progression of established Chagas cardiomyopathy (6–8). Data on treating adults with chronic disease are less established (3, 6, 10, 14). The CDC recommends offering antiparasitics to individuals without cardiac involvement from the ages of 18–50 years. There are no data to show that prophylactic antitrypanosomal therapy decreases the risk for reactivation for seropositive transplant patients, and given the high likelihood of side effects, it is not recommended (7). With prospective monitoring in the transplant population, most reactivation cases are detected, and preemptive treatment with benznidazole is initiated before symptoms and detection of the parasite on smear with good outcomes (6). Similar protocols for managing Chagas disease in patients with lymphoma and leukemia should be considered.

### Conclusion

As Chagas disease remains a largely neglected tropical disease, there is limited awareness of the severity and chronicity of infection outside of Latin America. However, with increased migration and globalization, Chagas disease has become a worldwide medical consideration. This case highlights the importance of healthcare providers throughout the world being able to recognize, diagnose, and treat Chagas disease. Screening at-risk patients for Chagas disease who are scheduled to undergo immunosuppression is imperative, and continued monitoring with molecular tools for reactivation is essential.
